# Combined oropharyngeal nasal (ON) swabs for the molecular detection of respiratory pathogens including *M. pneumoniae* in symptomatic children

**DOI:** 10.1128/spectrum.02181-25

**Published:** 2025-09-12

**Authors:** Hannah Nelson, Iryna Kayda, Nicole Watson, Mai-Lei Woo Kinshella, Jennifer Cochrane, Neil Desai, Michelle Dittrick, Linda Hoang, Michael T. Hawkes, Garth Meckler, Vikram Sabhaney, Jonathan B. Gubbay, Miguel Imperial, Jocelyn A. Srigley, Lynne Li, David M. Goldfarb

**Affiliations:** 1 Experimental Medicine Graduate Program, University of British Columbia, Vancouver, British Columbia, Canada; 2 BC Children’s Hospital Research Institute, Vancouver, British Columbia, Canada; 3 Department of Pathology and Laboratory Medicine, BC Children’s and Women’s Hospital & Health Centre, Vancouver, British Columbia, Canada; 4 Department of Obstetrics and Gynecology, University of British Columbia, Vancouver, British Columbia, Canada; 5 Community Based Testing and Biomedical Initiatives, First Nations Health Authority, Vancouver, British Columbia, Canada; 6 Department of Pediatrics, University of British Columbia, Vancouver, British Columbia, Canada; 7 Public Health Laboratory, British Columbia Centre for Disease Control, Vancouver, British Columbia, Canada; 8 Department of Pathology and Laboratory Medicine, University of British Columbia, Vancouver, British Columbia, Canada; Nationwide Children's Hospital, Columbus, Ohio, USA

**Keywords:** microbiology, virology, molecular diagnostics, pediatric infectious disease, parent-collected swab, self-collected swab, *Mycoplasma pneumoniae*, respiratory virus, nasopharyngeal swab, oropharyngeal nasal swab

## Abstract

This study aimed to evaluate the diagnostic yield and acceptability of parent-/caregiver-collected oropharyngeal nasal (ON) swabs compared with healthcare worker (HCW)-collected nasopharyngeal (NP) swabs for detecting respiratory viruses and *Mycoplasma pneumoniae* in children. Symptomatic children (0–4 years) presenting to the BC Children’s Hospital Emergency Department provided HCW-collected NP and caregiver-collected ON swabs. Acceptability was assessed using a 5-point Likert scale. Samples were tested using the SARS-CoV-2/Influenza A + B/RSV GeneXpert assay, and a subset was tested on the BioFire respiratory panel (RP) 2.1 during the initial research phase. In a subsequent implementation phase during a high incidence of *M. pneumoniae*, paired ON and NP swabs were collected and tested on the RP 2.1 in a quality improvement initiative. All *M. pneumoniae*-positive samples underwent testing for cycle threshold values. We analyzed 139 pairs from the research phase and 219 from the implementation phase. Detection rates for SARS-CoV-2, influenza A/B, and RSV were similar across swab types. Of 273 pairs tested on BioFire, ON swabs demonstrated significantly higher detection of *M. pneumoniae* (sensitivity of 94%, confidence interval [CI] [86, 98] ON vs 64% [61–75] NP, *P* = 0.0020) and comparable detection for other pathogens. Caregivers rated ON swab collection as more acceptable than NP swabs (median 4.5 [4–5] vs 2 [IQR 1–3], *P* < 0.0001). ON swab performance was similar to NP swabs for the detection of respiratory viruses and showed improved diagnostic yield of *M. pneumoniae*. Given their higher acceptability ratings, ON swabs should be considered as a less-invasive diagnostic option for children.

**IMPORTANCE:**

The current clinical standard for diagnosing respiratory infections in children typically requires a nasopharyngeal (NP) swab, which may be uncomfortable for the patient and must be collected by a healthcare worker. This study presents a less-invasive option, an oropharyngeal nasal (ON) swab collected by a parent or caregiver, that has comparable detection for common respiratory viruses and improved detection for *Mycoplasma pneumoniae*, a common but treatable cause of childhood pneumonia. Additionally, parents/caregivers rated ON swabs as significantly more acceptable than NP swabs on a scale of 1 to 5. Given the increased acceptability and superior detection of treatable *M. pneumoniae*, ON swabs are a patient-centered alternative to NP swabs and should be considered as a preferred diagnostic option for children.

## INTRODUCTION

The COVID-19 pandemic led to an increased demand for respiratory testing globally (1), as well as demand for more acceptable sample collection methods. The current clinical standard for diagnosis of all respiratory viruses is a healthcare worker (HCW)-collected nasopharyngeal (NP) swab (2, 3). NP swab collection is frequently uncomfortable for the patient (4–6) and may be exacerbated in children who interpret the discomfort as pain, leading to difficulties obtaining high-quality samples (7, 8). The fear of pain may result in avoiding NP swab collection altogether (9). Instead, we sought to explore the use of a combined oropharyngeal nasal (ON) swab collection method. This less-invasive sampling method can be collected by the parent/caregiver using the same flocked swab used for NP sample collections. ON swabs have been evaluated for COVID-19 detection (10); however, no previous work has identified its diagnostic performance for other respiratory pathogens.

A global resurgence of *Mycoplasma pneumoniae* (11) has prompted further evaluation of oropharyngeal swabs for respiratory diagnostics. *M. pneumoniae* is a common but treatable cause of pneumonia, particularly in children, and accurate diagnostics are needed to guide proper treatment (12–14). We adapted ON sample collection instructions previously utilized for COVID-19 diagnosis in Canada (10, 15) and investigated its performance compared with standard NP swabs for respiratory diagnostic testing in children.

## MATERIALS AND METHODS

### Research phase

Participants with acute respiratory infection symptoms (0–4 years old) were recruited from a pediatric Emergency Department (ED) where they were undergoing clinical testing with a HCW-collected NP swab during the 2022/2023 or 2023/2024 respiratory season.

Once consented, the participant provided an additional ON swab collected by the parent/caregiver prior to ED discharge using written self-collection instructions (Fig. S1) adapted from COVID-19 testing, as previously described (10, 15). Both the ON and NP samples were collected with the Copan FLOQSwab and immediately placed in Copan Universal Transport Medium. After both the ON and NP samples were collected, the parent/caregiver was asked to rate the acceptability of each method using a 5-point Likert scale: 5 being “acceptable,” 4 being “slightly acceptable,” 3 being “neutral,” 2 being “slightly unacceptable,” and 1 being “unacceptable.”

The specimens were transported to the BCCH Virology Lab where the NP swab sample was tested with the BioFire RP2.1 respiratory panel (RP 2.1; BioFire Diagnostics, LLC, Salt Lake City, UT, USA). Both the NP swab and ON swab samples were tested using the GeneXpert SARS-CoV-2/Influenza A + B/RSV assay (Xpert Xpress; Cepheid, Inc., Sunnyvale, CA, USA; Cepheid, Inc., Sunnyvale, CA, USA) according to the manufacturer’s instructions. A portion of the ON swab samples was also tested with the BioFire RP2.1 respiratory panel to capture a wider range of viral targets. All samples were tested the same day or stored promptly at −80°C and thawed to room temperature, then vortexed prior to testing.

### Implementation phase

During a *M. pneumoniae* resurgence in the 2024 respiratory season, ON swabs were implemented in the BCCH ED as part of a quality improvement project. A total of 219 symptomatic children who met clinical testing criteria (16) provided both an ON and NP swabs in accordance with guidance from the US Centers for Disease Control and Prevention (CDC) that recommended that clinicians consider sampling both the nasopharynx and the throat for improved *M. pneumoniae* detection (https://www.cdc.gov/mycoplasma/hcp/clinical-overview/index.html). During this period, both sample types were tested on the BioFire RP2.1 respiratory panel as part of routine clinical care. All samples with detection of *M. pneumoniae* underwent additional testing with a previously validated laboratory-developed polymerase chain reaction (PCR) test that targets the *M. pneumoniae* P1 cytadhesin gene (17), which also provided cycle threshold (Ct) values.

### Data analysis

Pathogen detection for ON and NP swab samples was compared with both the GeneXpert assay and BioFire panel. For the sensitivity calculation, a composite reference standard was set as testing positive on either ON or NP swab sample as all tested children had compatible respiratory symptoms that justified testing. Target pathogens with six or more positive detections underwent sensitivity calculation. A combined all-treatable pathogen targets (SARS-CoV-2, influenza A, influenza B, *Bordetella pertussis, Chlamydia pneumoniae,* and *M. pneumoniae, Bordetella parapertussis*), and all viral combined targets (SARS-CoV-2, influenza A, influenza B, RSV, adenovirus, coronavirus 229E, coronavirus HKU1, coronavirus NL63, coronavirus OC43, human Metapneumovirus, human Rhinovirus/Enterovirus, parainfluenza virus 1, parainfluenza virus 2, parainfluenza virus 3, and parainfluenza virus 4) were also assessed. Analyses and data visualization were performed using R (version 4.3.0) and GraphPad Prism (GraphPad Software, Boston, Massachusetts USA, version 10.2.0).

Descriptive statistics used the number and percentage for binary data, and median and interquartile range (IQR) for continuous data. To compare the sensitivity of the ON and the NP swabs, we used McNemar’s test. We corrected for multiple comparisons using the Bonferroni method. For comparison of paired Ct values, we used the Wilcoxon signed ranks test. For comparison of paired acceptability scores (NP swab and oral/nasal swab), we classified responses as: NP swab score higher, ON swab score higher, or tie, and used McNemar’s test to compare the paired scores. For the comparison of the viral targets, if one of the targets was negative, Ct was set to 40 for calculations. To compare Ct values across multiple targets between the ON and NP swabs, we modeled the Ct value as a function of the swab type using a linear mixed effects (LME) model (package *lmer* in the R statistical environment), adjusting for the target pathogen (fixed effect), interaction between target and swab type (fixed effects), and individual patient (random effect).

## RESULTS

**Fig 1:**
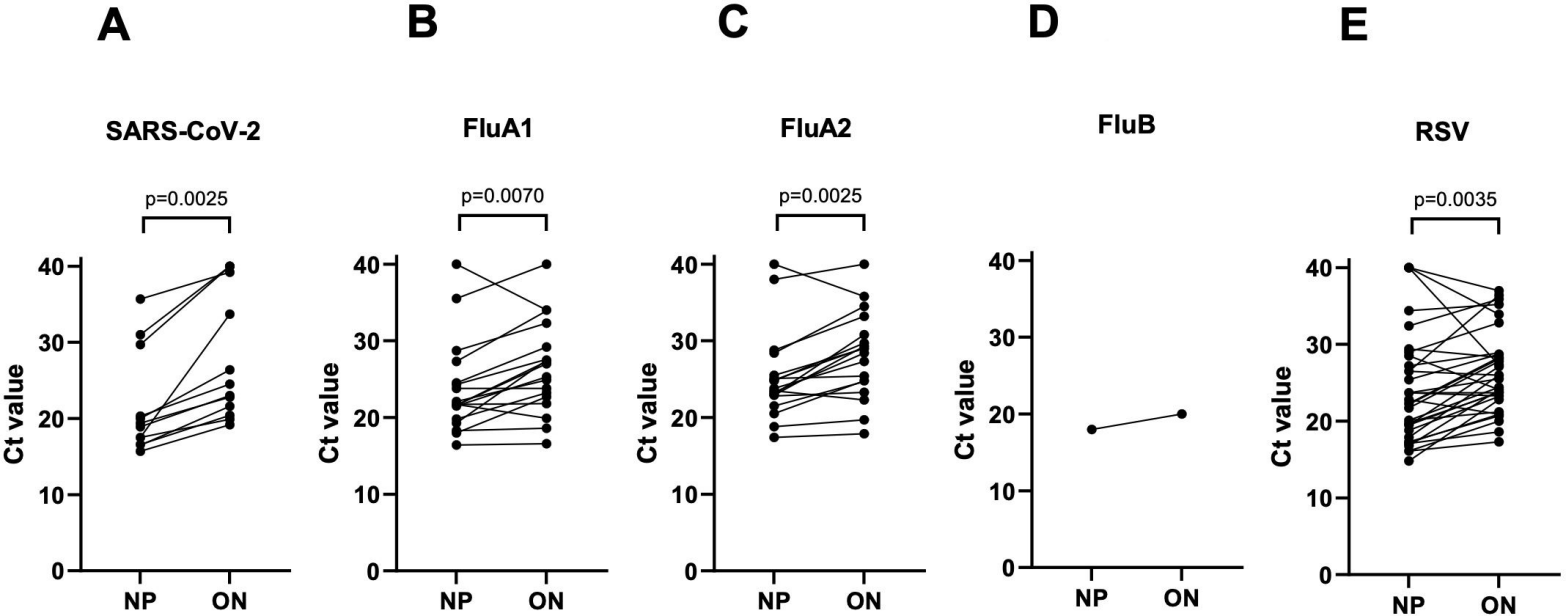
Paired Ct values from nasopharyngeal (NP) and oropharyngeal nasal (ON) swabs. (**A**) SARS-CoV-2, (**B**) influenza A1 target, (**C**) influenza A2 target, (**D**) influenza B, and (**E**) RSV using the GeneXpert assay. Data are shown for paired samples for which at least one of the swabs tested positive.

A total of 139 matched sample pairs were collected and tested with the GeneXpert SARS-CoV-2/Influenza A + B/RSV assay in the research phase between January to April 2023 and November 2023 to March 2024. The median age of participants was 16 months old (IQR 8–31), with 63% male participants. Given that only one case of influenza B was detected during the study period, it was combined with influenza A for sensitivity calculation. The ON swab detected 61 total target pathogens on the GeneXpert assay, and the NP swab detected 60. Coinfections were seen in 3 out of 139 (2%) sample pairs. Two samples involved coinfection of influenza A and RSV; however, in one of the two sample pairs, RSV was not detected on the NP swab. A total of 61 (44%) of the 139 participants tested positive for at least one of the target pathogens on either the ON or NP swab, and 54 (39%) tested positive on both swabs. All matched ON and NP swab pair testing results with the GeneXpert assay are shown in Table 1. Among paired samples that were positive from either ON or NP swab, the median (IQR) Ct values were higher for the ON swab (Table 2; Fig. 1). In an LME model considering Ct values for all targets, the ON swab had a Ct value 2.7 cycles (95% CI 2.4, 3.2) higher, on average, than the NP swab (*P* < 0.0001). Of the 139 matched sample pairs tested with the GeneXpert assay in the research study phase, 54 pairs were also tested using the BioFire RP2.1 respiratory panel and showed a similar yield for total respiratory pathogens detected (63 ON swab detections vs 62 NP swab detections). Samples tested on the RP2.1 panel were the most recent 54 at the time of testing. An additional 219 samples were collected and tested on the BioFire panel between 26 November and 28 December 2024 during the implementation phase. The initial 54 samples tested were combined with the additional 219 to provide data for 273 matched pairs tested in the BioFire panel. The mean age of participants was 6 years and 2 months. All matched ON and NP swab pair testing results with the BioFire panel are shown in Table 3, with target pathogens individually shown if they had six or more detections. Notably, ON swabs had better detection for *M. pneumoniae* when compared with NP swabs. All viral combined pathogen target detection was not significantly different between sample types.

**TABLE 1:** Comparison of viral target detection in 139 matched swab sample pairs using GeneXpert SARS-CoV-2/Influenza A + B/RSV assay^*c*^

Pathogen target	Either sample positive	Both sample positive	ON swab positive only	NP swab positive only	Both samples negative	ON swab sensitivity (%) [95% CI]^*a*^	NP swab sensitivity (%) [95% CI]^*a*^	McNemar *P*
SARS-CoV-2	12	10	0	2	127	83 (52–98)	100 (74–100)	0.48
Influenza^*b*^	19	17	1	1	120	95 (74–100)	95 (74–100)	>0.99
RSV	33	30	3	0	106	100 (89–100)	91 (76–98)	0.25

^*a*^ Either sample positive is set as the reference for the calculation for sensitivity.

^*b*^ Influenza A1 target, Influenza A2 target, or Influenza B target positive.

^*c*^ ON, oropharyngeal nasal; NP, nasopharyngeal.

**TABLE 2:** Paired Ct values from oropharyngeal nasal (ON) and nasopharyngeal (NP) swabs using the GeneXpert assay^*e*^

	N^*a*^	ON Ct value (95% CI)	NP Ct value (95% CI)	*P*-value^*b*^
SARS-CoV-2	12	24 (21–35)	19 (17–23)	0.0025
Flu A1**^*c*^**	18	26 (23–29)	22 (20–24)	0.007
Flu A2**^*c*^**	18	29 (25–31)	24 (23–25)	0.0025
Flu B^*d*^	1	20	18	–^*f*^
RSV	33	26 (23–28)	22 (19–27)	0.0035

^*a*^ Either sample positive.

^*b*^ All differences significant after Bonferroni correction at the α = 0.05/5 level of significance.

^*c*^ Two targets for Influenza A (A1 and A2) showed a high degree of correlation. The correlation coefficients were r = 0.99 between the Ct values for the FluA1 and FluA2 targets using the ON swab and r = 0.99 between the FluA1 and FluA2 targets using the NP swab.

^*d*^ Only one patient had a positive sample for influenza B, unable to calculate a *P*-value.

^*e*^ ON, oropharyngeal nasal; NP, nasopharyngeal.

^*f*^ “–”, Not calculated due to small number of detections.

**TABLE 3:** Comparison of pathogen detection in 273 matched swab sample pairs using the BioFire RP2.1 respiratory panel with sensitivity calculations for target pathogens with *n* > 6^*f*^

Pathogen target	Either sample positive	Both sample positive	ON swab positive only	NP swab positive only	Both samples negative	ON swab sensitivity (%) [95% CI]^*a*^	NP swab sensitivity (%) [95% CI]^*a*^	McNemar *P*
Adenovirus	24	11	6	7	249	71 (49-87)	75 (53–90)	>0.99
Corona 229E	0	0	0	0	273	–^*g*^	–	–
Corona HKU1	2	2	0	0	271	–	–	–
Corona NL63	2	1	1	0	271	–	–	–
Corona OC4A	2	2	0	0	271	–	–	–
SARS-CoV-2	7	6	0	1	266	86 (42–100)	100 (59–100)	>0.99
Human Metapneumovirus	19	17	1	1	254	95 (74–100)	95 (74–100)	>0.99
Human Rhinovirus/ Enterovirus	47	27	10	10	226	79 (64–89)	79 (64–89)	>0.99
Influenza A	25	21	3	1	248	96 (80–100)	88 (69–97)	0.62
Influenza B	5	4	0	1	268	–	–	–
Parainfluenza 1	9	7	0	2	264	78 (40–97)	100 (66–100)	0.48
Parainfluenza 2	5	2	2	1	268	–	–	–
Parainfluenza 3	2	1	0	1	271	–	–	–
Parainfluenza 4	10	4	1	5	263	50 (19–81)	90 (55–100)	0.22
RSV	96	91	3	2	177	98 (93–100)	97 (91–99)	>0.99
*Bordetella parapertussis*	0	0	0	0	273	–	–	–
*Bordetella pertussis*	1	0	1	0	272	–	–	–
*Chlamydia pneumoniae*	1	1	0	0	272	–	–	–
*Mycoplasma pneumoniae* **^*b*^**	69	40	25	4	204	94 (86–98)	64 (51–75)	0.00020^*e*^
All treatable combined **^*c*^**	97	65	27	5	176	95 (88–98)	72 (62–81)	0.00021^*e*^
All viral combined **^*d*^**	194	174	10	10	79	95 (91–98)	95 (91–98)	>0.99

^*a*^ Either sample positive is set as the reference for the calculation for sensitivity.

^*b*^ The total number of positive specimens was 65 using the ON swab and 44 using the NP.

^*c*^ All-treatable pathogen targets combined includes SARS-CoV-2, influenza A, influenza B, *Bordetella pertussis, Chlamydia pneumoniae,* and *Mycoplasma pneumoniae*.

^*d*^ All viral combined includes all the viral BioFire RP2.1 respiratory panel viral targets: SARS-CoV-2, Flu A, Flu B, RSV, Adenovirus, Coronavirus 229E, Coronavirus HKU1, Coronavirus NL63, Coronavirus OC43, Human Metapneumovirus, Human Rhinovirus/Enterovirus, Parainfluenza Virus 1, Parainfluenza Virus 2, Parainfluenza Virus 3, and Parainfluenza Virus 4.

^*e*^ Significant at the α = 0.05/22 level of significance.

^*f*^ ON, oropharyngeal nasal; NP, nasopharyngeal.

^*g*^ “–”, Not calculated due to small number of detections.

**Fig 2:**
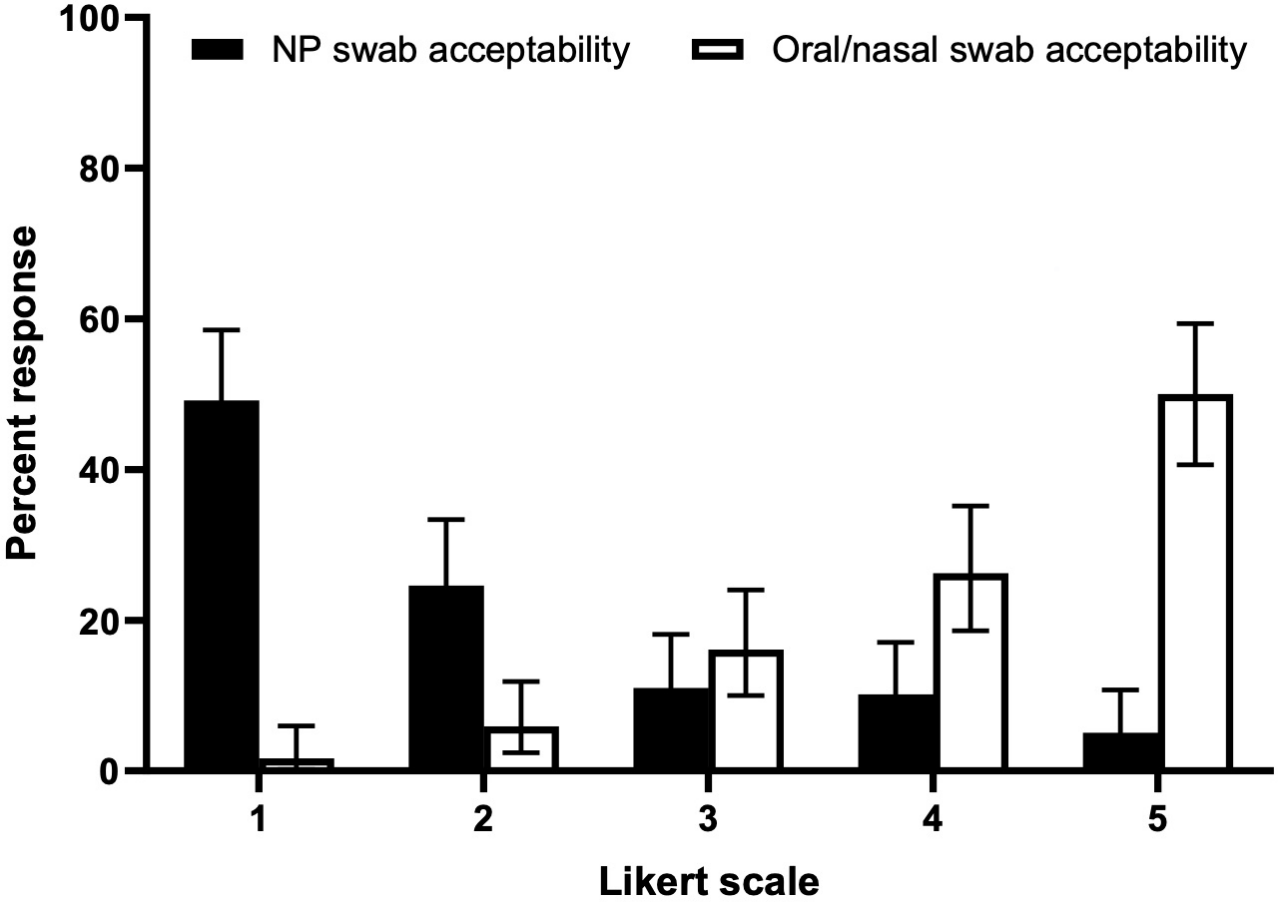
Acceptability ratings for 118 matched swab sample pairs. Respondents ranked the acceptability of both the nasopharyngeal (NP) swab (black bars) and oropharyngeal nasal (ON) swab (white bars) on a Likert scale from 1 (least acceptable) to 5 (most acceptable). Bars indicate the percent of responses, and the whiskers represent the binomial 95% CI. The median acceptability scores of the NP swab and oral/nasal swab were 2 (IQR 1–3) and 4.5 (4–5), respectively (*P* < 0.0001).

The median Ct values for matched samples with at least one detection of *M. pneumoniae* provided by a laboratory developed test are shown in Table 4. Of the 69 *M*. *pneumoniae detections,* matched sample results were excluded if either sample result did not have enough sample volume (*n* = 8), or if the result was indeterminate (samples that were negative on laboratory-developed PCR test but positive on BioFire; *n* = 11). In both the overall group and the group with matched *Mycoplasma pneumoniae* positives, the difference in Ct value was not statistically significant (Table 4). The acceptability scores for 118 matched swab sample pairs were provided by parent or caregivers of the participant during the study phase, and the results are shown in Fig. 2. The median acceptability scores of the NP swab and oral/nasal swab were 2 (IQR 1–3) and 4.5 (IQR 4–5), respectively (*P* < 0.0001).

**TABLE 4:** Median cycle threshold (Ct) values for matched samples with at least one detection of *Mycoplasma pneumoniae* and Ct value differences by laboratory-developed test^*a*^

*Mycoplasma* pneumoniae positive (*N*)	ON swab Ct Median (IQR)	NP swab Ct median (IQR)	Difference in Ct value Median (IQR)	*P*-value
ON and NP Swab both positive (*n* = 27)	32 (29–35)	33 (30–35)	1.4 (−2.4 to 4.3)	0.44
ON swab positive only (*n* = 19)	34 (31–35)			
NP swab positive only (*n* = 4)		34 (32–37)		
Overall (*n* = 50)	32 (29–35)	33 (30–36)	1.4 (−2.4 to 4.3)	0.44

^*a*^ ON, oropharyngeal nasal; NP, nasopharyngeal.

## DISCUSSION

This study demonstrated that parent/caregiver-collected ON swabs had significantly better detection for *M. pneumoniae* when compared with HCW-collected NP swabs. For viruses, such as SARS-CoV-2, influenza A/B, and RSV, ON swabs had comparable diagnostic performance to NP swabs for detecting respiratory viruses in children. Additionally, ON swabs were rated by families as significantly (*P* < 0.0001) more acceptable than NP swabs, suggesting they may have potential to be a less invasive diagnostic option for pediatric populations.

Although the CDC recommends consideration for sampling of both the nasal and oropharyngeal areas for diagnosis of *M. pneumoniae* infections, the ON sample type may eliminate the need for two separate swabs to be collected and tested if viral testing is also needed. Other studies also suggest that pharyngeal swabs have improved sensitivity for *M. pneumoniae* than NP swabs (18, 19); however, oropharyngeal swabs generally have lower yield for viruses (20).

This study demonstrated that on both the GeneXpert assay and the BioFire RP2.1 panel, ON swabs exhibited similar viral pathogen detection rates to NP swabs (Tables 1 and 2). The higher overall Ct values for ON swabs may indicate a lower viral load in these samples, but these data show it did not impact their clinical sensitivity. The all-treatable combined target also reflected significant difference; however, this sample population was driven by 71% *M*. *pneumoniae* samples.

Another study that compared detection rates for a range of viral pathogens in saliva samples, nasal samples, and NP swabs independently found that NP swabs had a higher rate of detection, with higher viral load overall (12–14). This is in line with another study that found alternate sampling methods, such as nasal swabs or saliva samples, inferior to NP swabs (21). Based on prior studies conducted in response to the COVID-19 pandemic, combined swabbing of throat and nose for ON samples yielded better clinical sensitivity when compared with other methods (22). Further, combined ON swabs have been implemented for SARS-CoV-2 molecular testing in both Canada and the UK (15, 23).

An important finding was the higher acceptability of ON swab collection among parents/caregivers. The lower acceptability of NP swabs can likely be attributed to their invasive nature, as they require deep insertion into the nasopharynx, causing discomfort. This is consistent with prior literature suggesting that NP swab collection is more painful than other less invasive methods (8). Importantly, over a quarter of parents identified concern for pain associated with NP collection as a reason to avoid testing (24), and the higher acceptability of ON swabs may mitigate those concerns. ON swabs offer a method that can be self- or parent/caregiver-collected for children, is less invasive, and may reduce distress for both children and parent/caregiver. This is important from a clinical standpoint as it is difficult to distinguish *M. pneumoniae* from other pathogens based on clinical and x-ray findings alone. Self-collection could also potentially reduce the burden on HCWs, who are required to collect NP swabs, freeing physical resources such as personal protective equipment, as well as human resources in locations with high testing demand. Additionally, ON swabs may facilitate testing in settings where cultural or logistical barriers exist, such as remote or Indigenous communities, where NP swabbing might be less accessible/acceptable.

This study design had several strengths, including robust comparison using two common commercial assays (GeneXpert & BioFire RP2.1) and inclusion of numerous viral targets and bacterial targets. As far as we are aware, this is the largest study to compare sample collection methods for *M. pneumoniae* diagnosis. Conveniently, the same flocked swab type was used for both samples, and therefore ON swabs do not require the establishment of new supply chain to implement in our local settings.

Overall, this study demonstrated parent-/caregiver-collected ON swabs had significantly better detection of *M. pneumoniae* when compared with HCW-collected NP swabs. The performance of ON swabs was comparable to NP swabs for the detection of a range of respiratory viruses across two commercial molecular assays. Given the significantly higher acceptability ratings and improved sensitivity for the detection of clinically relevant pathogens, ON swabs should be considered as a preferred sample and a less-invasive diagnostic option for children in the pediatric ED.

## SUPPLEMENTAL MATERIAL

Fig. S1 spectrum.02181-25-s0001.pdf Combined oral/nasal swab sample collection instructions.

ASM does not own the copyrights to Supplemental Material that may be linked to, or accessed through, an article. The authors have granted ASM a non-exclusive, world-wide license to publish the Supplemental Material files. Please contact the corresponding author directly for reuse.
