# Supplementary material for: Combined oropharyngeal nasal (ON) swabs for the molecular detection of respiratory pathogens including M. pneumoniae in symptomatic children
Source: Microbiol Spectr. 2025 Sep 12;13(10):e02181-25. doi: 10.1128/spectrum.02181-25 (PMC12502526; doi:10.1128/spectrum.02181-25)

Combined Oral/Nasal Swab Sample Collection Instructions

For testing accuracy please DO NOT eat, drink (including water), brush teeth, or chew gum for 15 min prior to testing.

1. Both you and the child wash hands with soap and water or hand sanitizer and place a mask on yourself.

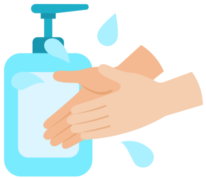

2. Carefully open swab package and take out the swab by the stick part. **Avoid touching the soft tip of the swab.**

3. **Oral (MOUTH):** Gently place the soft tip of the swab into your child's mouth.

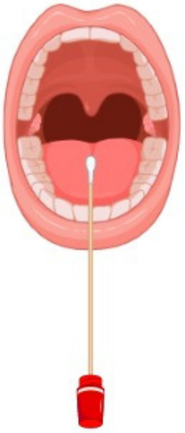

1. Place onto the back of the tongue touching the the throat, gently rub the swab in circles for 5 seconds.
2. Move swab to inside of cheek, and gently rub the swab in circles for 5 seconds.
3. Move to inside of other cheek, and gently rub in circles for 5 seconds.
4. Remove swab from mouth.

4. **Nasal (NOSE):** Gently place the soft tip of the swab into your child's nose.

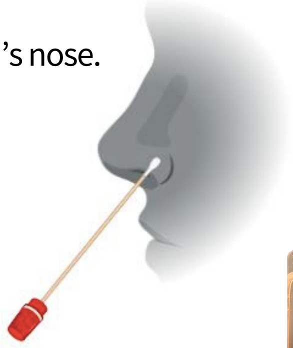

1. Slowly move the swab in circles and rub along the inside of the nostril for 5 seconds.
2. Move to other nostril, and gently rub in circles for 5 seconds.
3. Remove swab from nose.

5. Place collected swab into the tube containing liquid and holding the shaft close to the rim of the tube, and keeping the rim of the away from your face, break the applicator shaft and the red breakpoint indication line. Twist the cap tightly to close. Avoid drinking or spilling the liquid. Place the tube back into the biohazard bag and close ziplock

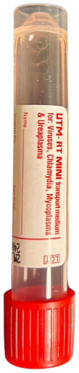

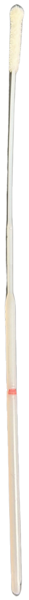

6. Both you and the child wash hands with soap and water or hand sanitizer. Please return the bag with your mouth/nose swab sample to the healthcare worker.

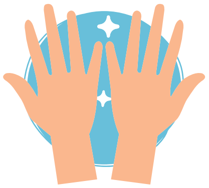

Supplement: Fig. S1 — Combined oral/nasal swab sample collection instructions. [file spectrum.02181-25-s0001.pdf]
